# PRo-Pat: Probabilistic Root–Pattern Bi-gram data language model for Arabic based morphological analysis and distribution

**DOI:** 10.1016/j.dib.2022.108875

**Published:** 2023-01-05

**Authors:** Bassam Haddad, Ahmad Awwad, Mamoun Hattab, Ammar Hattab

**Affiliations:** a Department of Data Science and Artificial Intelligence, University of Petra, Amman, Jordan; b Lewis University, Romeoville, IL 60446, USA; c Arabic Textware Company, Amman, Jordan; d Brown University, Providence, RI 02912, USA

**Keywords:** Arabic language model, Root Pattern Analysis, Word Cognition, N-gram models, Probabilistic morphology, Root-Pattern Classification

## Abstract

Based on 29,192,662 html files obtained from the ClueWeb a bi-gram data language model for Arabic is constructed. The created dataset is considering standard types of bi-gram analysis, however with focus on the root^1^-pattern^2^ paradigm in Arabic. Root-Pattern distributions in form of P(root|pattern), P(pattern|root) and P(pattern|pattern) are additionally estimated. The aspect of considering the Maximum Likelihood Estimation (MLE) on the root-pattern level as a higher-level of abstraction, has been widely neglected in Arabic research community despite its advantage in reducing ambiguities within Arabic morphological analysis and its impact on cognitive aspect on Arabic word perception [1]. In the preprocessing phase, the html files were converted to 974 unfiltered raw text files with the size of about 180 GB. These files were morphologically analyzed towards extracting and counting frequencies of patterns, roots, particle, and stems and particularly root-pattern occurrences. Based on a resulting corpus containing around 18,482,719 raw words, a language data model is constructed containing 9,311,246 bi-grams of morphologically analyzed wordform, including around 3.49 million bi-directional P(root|pattern) and around 1.153 million P(patttern|pattern) bi-grams in form of conditional probabilities covering a subset of around 8086 roots with 20413 possible pattern-forms. As this data model is considering the root–pattern phenomenon in Arabic, the created data are useful for researchers working on cognitive aspect of Arabic such as visual word cognition, morpho-phonetic perception, morphological analysis, spell-checking, and resolving ambiguities in morphological parsing.

**Specifications Table**

**Table:** 

Subject	Artificial Intelligence
Specific subject area	Natural Language Processing / Probabilistic Language Data Model based bi-gram analysis.
Type of data	7 Files tables including 3,496.012 bi-gram records representing Maximum Likelihood Estimations. Raw text corpus contains unfiltered text files extracted from 974 WARC files with the size of about 180 GB.
How the data were acquired	29,192,662 html webpages of Clueweb were extracted and converted into 974 raw unfiltered text files; each one contains 30,000 initial webpages in average. These text files were then morphologically analyzed and indexed. Different morphological analyzers were used (Arabic Textware and Alkalil morphological analyzers, and Khoja stemmer) to extract corpus stems, roots and patterns. To estimate frequency of occurrence and conditional probabilities, a tagged terms matrix was created to count smoothed occurrences of (root-root, root-pattern, pattern-pattern).
Data format	Raw Analyzed Filtered
Description of data collection	The initial Data (ClueWeb) was organized in a set of WARC archive files, from which the html files were extracted, parsed and then converted in text files. To prepare data for MLE, words were filtered from non-Arabic word (predominantly Persian Words), morphologically analyzed, and indexed in co-occurrence matrix.
Data source location	Web
Data accessibility	(1) PRo-Pat:The major Dataset is located on: Repositories name: **figshare,** • PRo-Pat: Root-Pattern Probabilistic Dataset: Data identification number: 10.6084/m9.figshare.21586326 Direct URL to data: https://figshare.com/s/1eecb1f53524a7a6d964 (2) Byproducts (supplemental) partially cleaned Datasets on:^3^ • Morphological Dataset Data identification number: 10.6084/m9.figshare.21604014 Direct URL to data: https://figshare.com/s/09524fbac079c5edf38e • Raw Text Corpus (Unfiltered partially cleaned Html Raw Text files) on Zenodo: Data identification number: https://doi.org/10.5281/zenodo.7349375 Direct URL to data: https://zenodo.org/record/7349376#.Y3510exBwlU
Related research article	B. Haddad, A. Awwad, M. Hattab and A. Hattab, "Associative Root–Pattern Data and Distribution in Arabic Morphology," *Data,* vol. 10, no. 3, pp. 2-17, 2018.

3 The “Morphological Dataset” and the “ Raw Text Corpus” are just supplemental byproducts to verify the extraction process of the major “PRo-Pat Dataset”. Our current model uses these texts as bag of words. We have added these additional datasets upon Journal requirement of making all data used in the process of creating the major Dataset available. However advanced deep cleaning of all textual Corpus can be followed as a future work. The text was just basically extracted from Html file and partially cleaned (around 30 million Html files). However, these raw data are still useful for many purposes. The major goal of this presentation is focused on presenting a missing dataset on a higher level of abstraction, namely, Root-Patters probabilistic data model, which have been successfully used in spell correction, Query-Expansion and Relevance Assessment.

## Value of the Data

• The main goal of a Language Model is to estimate the probability distribution of word sequences generated in a natural language such as Arabic. In statistical Natural Language Processing estimating probabilities is mainly based on Maximum Likelihood Estimation (MLE), which can be achieved by bi-gram analysis. In our Data Model, Arabic words sequence distribution were additionally considered from a cognitive point of view considering the singularity of the root–pattern phenomenon in Arabic word distributions [1,2]. This aspect allows us to estimate word sequences on a higher level of abstraction for words as root occurrences, and all their potential occurrences with multiple morpho-phonetic patterns [[5], [6]]. Predictions on P(root|pattern), P(pattern|root), (root|root) represent a higher level of abstraction than computing co-occurrences of their instances.
• The dataset provides natural language processing community with a predication model for Arabic language that is built on a representative large corpus [3]. The corpus contains 18,482,719 words gathered from 29,192,662 different html webpages.
• Additionally, this data model is useful in reducing ambiguines, particularly in non-determinism evolving in probabilistic bi-directional Root or Pattern extraction process. PRo-Pat probabilistic data represents in this connection an additional aid to rank possible root ambiguity based on maximal root estimation under certain patterns. For example, the maximal root or ranked root extraction can be estimated based on the following Bayesian classification model:
  $$\hat{R}=\underset{\mathrm{root}\in \mathrm{Root}}{\mathrm{argm}\mathrm{ax}}\;P\left(\mathrm{root}|\mathrm{patt}\mathrm{ern}\right)=\underset{\mathrm{root}\in \mathrm{Root}}{\text{argmax}}\;P\left(\mathrm{patt}\mathrm{ern}|\mathrm{root}\right)\;P\left(\mathrm{root}\right)$$

On the other hand, the backward estimation, i.e., the maximal or ranked pattern estimation under certain roots, can be estimated based on the following Bayesian classification model:

$$\hat{Pat}=\underset{\mathrm{pat}\in \mathrm{Patt}\mathrm{ern}}{\mathrm{argm}\mathrm{ax}}\;P\left(\mathrm{pat}|\mathrm{root}\right)=\underset{\mathrm{pat}\in \mathrm{Patt}\mathrm{en}}{\text{argmax}}\;P\left(\mathrm{root}|\mathrm{pat}\right)P\left(\mathrm{pat}\right)$$

These models provide researchers with supportive knowledge to resolve different types of ambiguities problems. Furthermore:

• N-gram of roots, stems, patterns language models might be an additional aid to instantiate surface level sentences where ambiguities might occur due to missing diacritics or predicting certain word sequences.
• Word similarly in context of spell error correction and Term translation [4].
• Representing word sequence as root-pattern associative network is useful in query expansion modeling and word-topic relevance assessment [[5], [6]].

## 1 Objective

The initial objective behind creating this dataset is to provide researchers with probabilistic Data to reduce ambiguities evolving in morphological and Part of Speech Tagging (POS) in context of improving spell checkers and search engines. These aspects were useful in improving performance of tools used by the researchers.

## 2 Data Description

The data model contains a corpus of 974 raw text files extracted from 29,192,662 ClueWeb html files stored 180 GB. Each text file contains in average 30,000 different webpages. The size of the corpus is 18,482,719 terms. The data are organized in 7 files tables, considering different unigram frequency of occurrence (root, pattern, stems) and their bi-gram MLE in form of conditional probabilities. The data model contains in addition, a morphological Database resulted in using three different morphological parser and stemmer (Arabic Textware and Alkalil morphological analyzers, and khoja stemmer).

The data model contains 9,311,246 bi-grams of morphologically analyzed wordform, with around 3.4 million bi-directional P(root|pattern) and around 1.153 million P(patttern|pattern) bi-grams in form of conditional probabilities covering around root 8086.

**Fig. 1:**
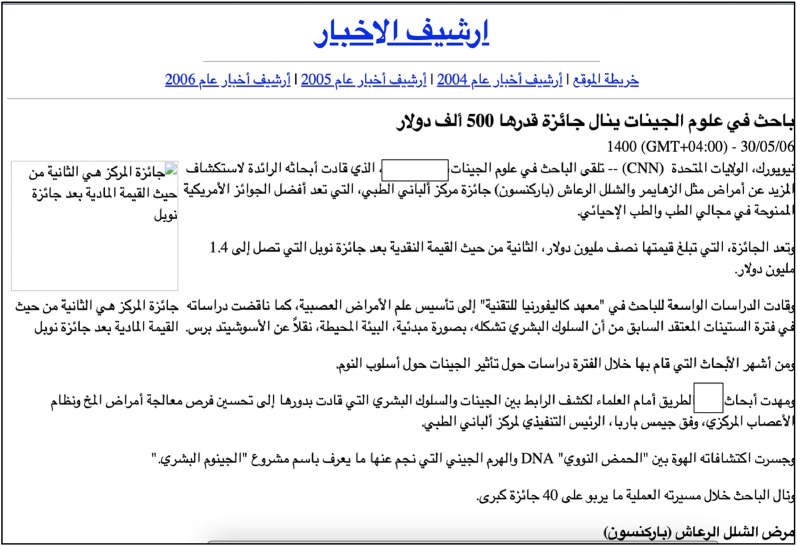
Sample of an initial raw ClueWeb html file before parsing and basic cleaning.

Fig. 1 shows a sample of initial-raw ClueWeb html file before parsing and basic cleaning. The number of the considered html files is around 29.2 million web pages. Content: *The title of this html file is “Archive News” (in bold blue)”. It contains website map, links to Archive new 2004, Archive 2005, and Archive 2006. The “news text” is talking about a genetic researcher reward in value of 500 thousand Dollar for his research in DNA and it influence on human behavior. See text translation:*

**Figure:**
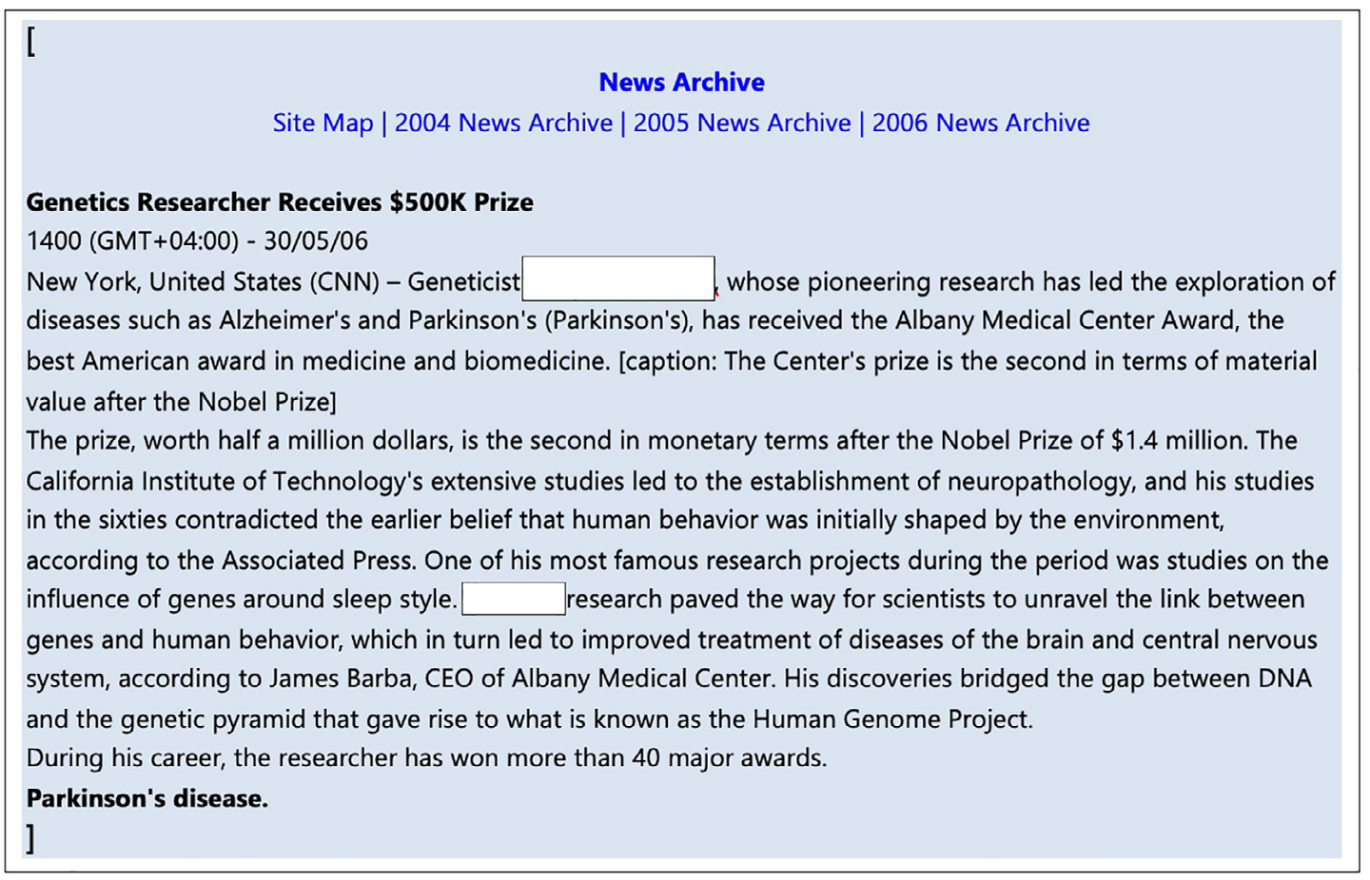
Text translation to English of figure 1.

**Fig. 2:**
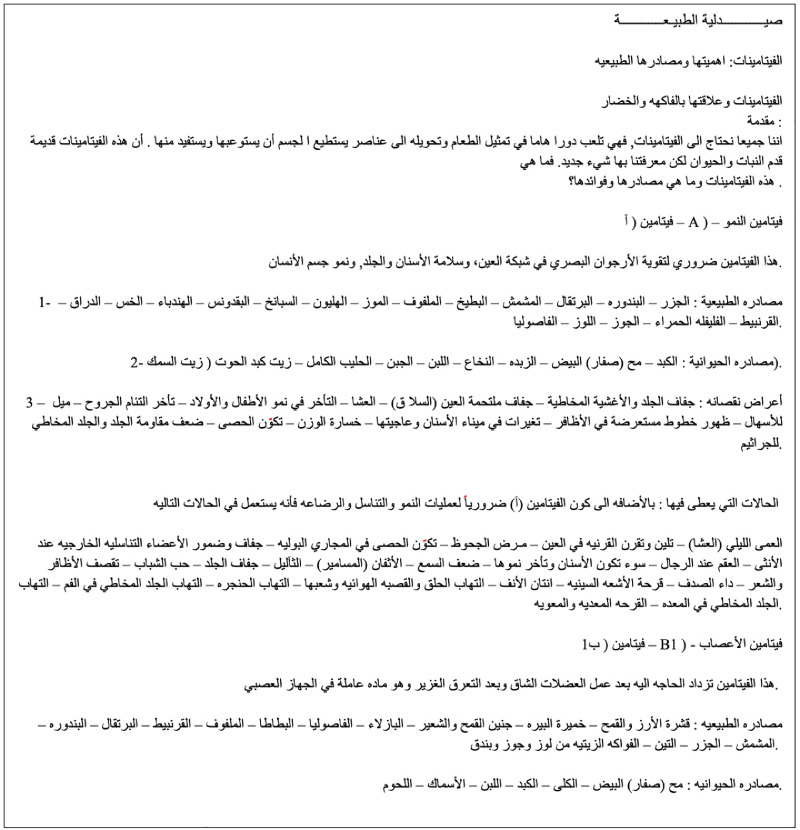
Sample of a fragment of an extracted of WARC file. These files are large, as they summarize the content of 30,000 webpage in average. See page 6.

Fig. 2 shows a sample fragment of an extracted WARC file. There are 974 file such these files. Each file contains in average 30,000 html files. Content: *The text is talking about physiotherapy and herbal treatment and vitamins. The advantage of different vitamins is list in the extracted text. Text translation:*

**Figure:**
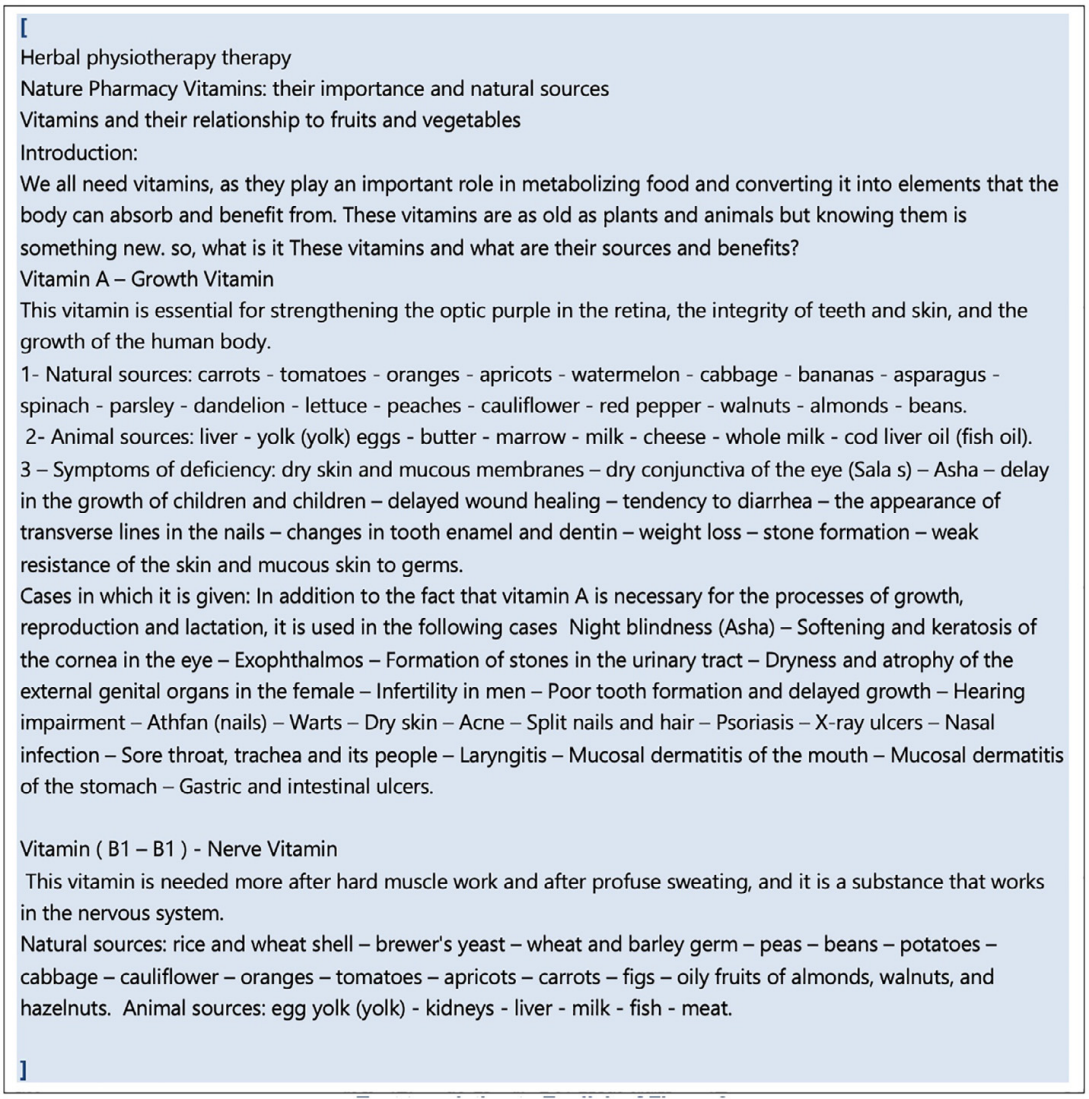
*Text translation to English of*Figure 2.

**Fig. 3:**
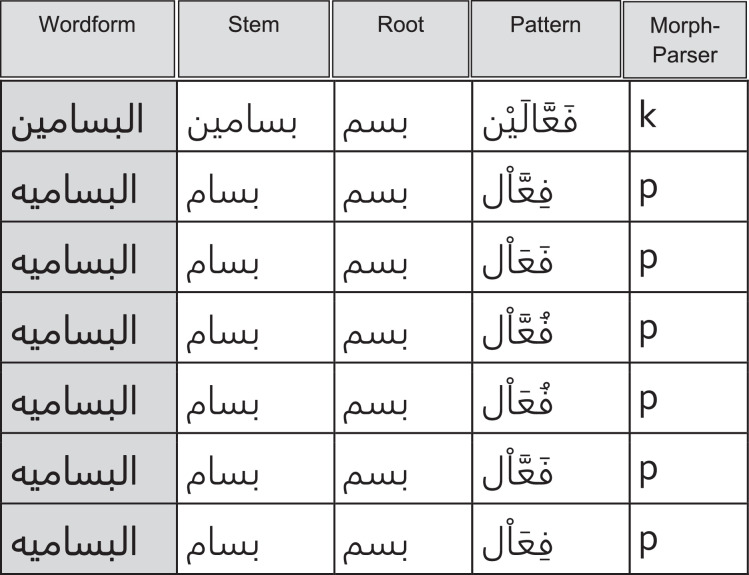
A fragment of morphological analysis using Arab Textware (p) and Alkahil (k) morphological Analyzers. A word form is reduced to a stem, root, and to a corresponding pattern. See page 13.

Fig. 3 shows a fragment of morphological analysis based on Arab Textware (p) and Alkahil morphological Analyzers. Content: *The table contains multiple records descripting the output morphological analysis performed by Arab Textware and Alkahil morphological Analyzers. For Example, the first record of the except shows the wordform: “alBaSSaMyn,” Stem: “bassamyn,” Root: “BSM”, Pattern: C_1_aC2C_2_*$\bar{a}$*C_3_yn, where C_1_, C_2_, and C_3_ are roots’ radicals. The root “BSM” is occurring in the templatic pattern: C_1_aC_2_C_2_*$\bar{a}$*C_3_yn as follows: C_1_ =B, C_2_=S, C_3_=M. The basic meaning of this root means smile. Analog are the rest records.*

**Fig. 4:**
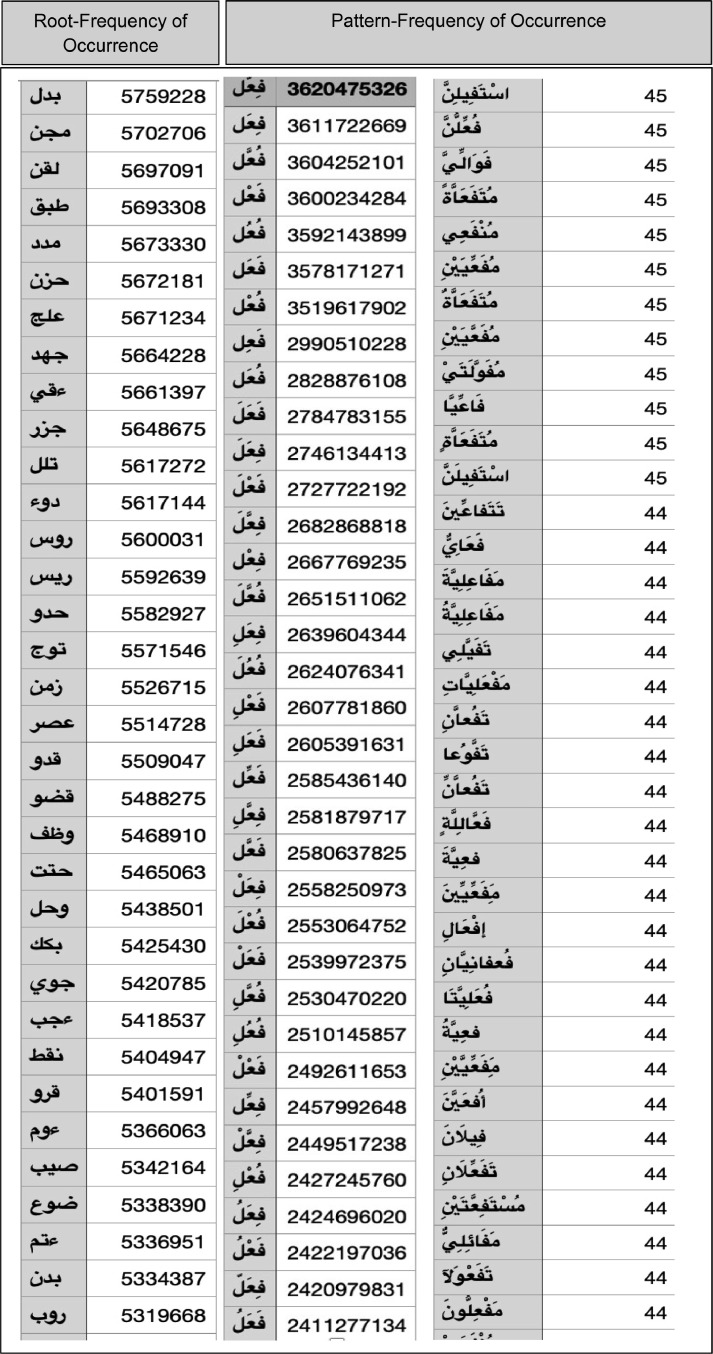
Sample of a fragment of roots, patterns and their frequency of occurrence towards estimating their corpus based MLE. 8065 roots and patterns with 20413 possible pattern-forms were considered. See page 13.

Fig. 4 shows a sample of a fragment of root, pattern frequency of occurrence towards estimating their corpus MLE. Content: *The table contains multiple records descripting the result of frequency counting for Arabic Roots and Patterns. For Example, the first record shows the root “BDL" with 579228 frequencies of occurrence. The basic meaning of this root is substitute. The frequency of the pattern “C1aC_2_C_2_C_3_“ is 3620475326, where C_1_=B, C_2_=D, and C_3_=l. Analog are rest records.*

**Fig. 5:**
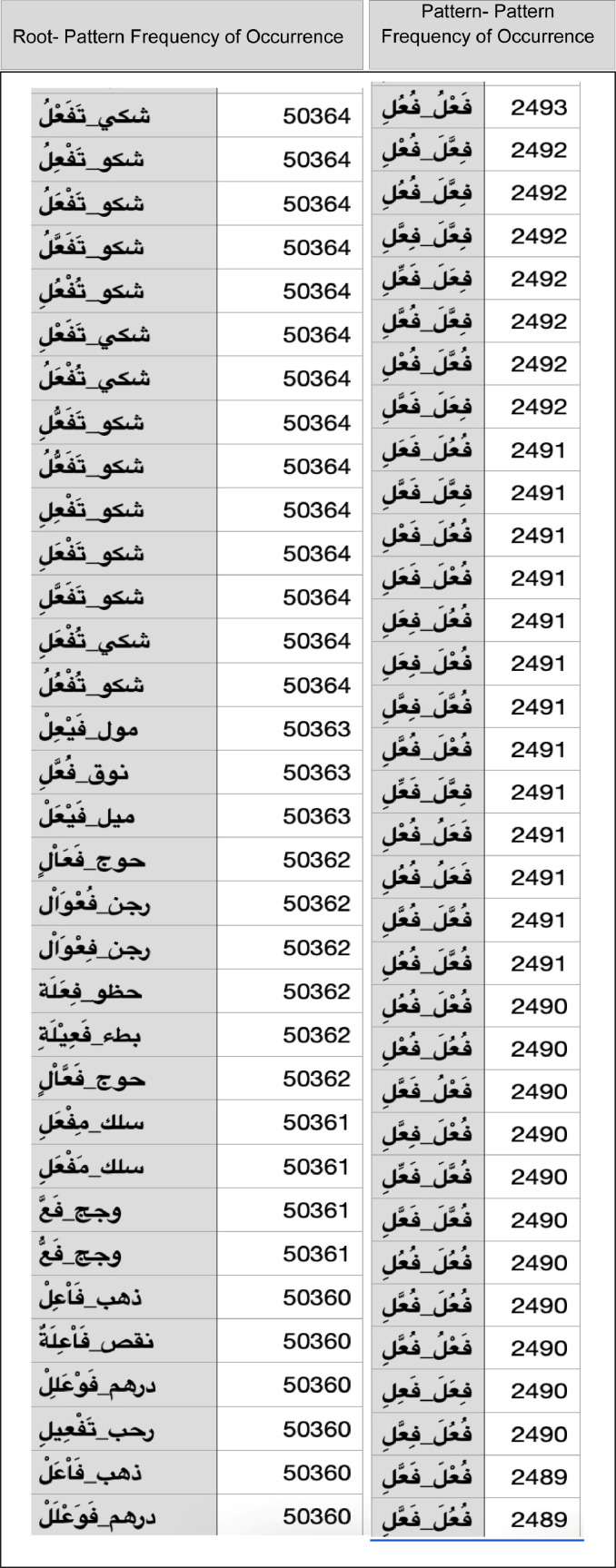
Sample of a fragment of Root-Pattern and Pattern-Pattern frequencies. See page 13.

Fig. 5 shows a sample of a fragment of root-pattern and pattern-pattern frequencies towards their MLE estimation. Content: *The table contains multiple records descripting the result of frequency counting for Arabic Root-Pattern co-occurrence. For Example, the first record shows the frequency of occurrence for the: ŠKY - taC_1_aC_1_aC_3_u with 50364. ŠKY basic meaning is “doubting”. And Pattern-Pattern frequency of occurrence for instance “C_1_aC_2_C3u - C_1_uC_2_C_3_i “ is 2493. Analog are the rest records.*

**Fig. 6:**
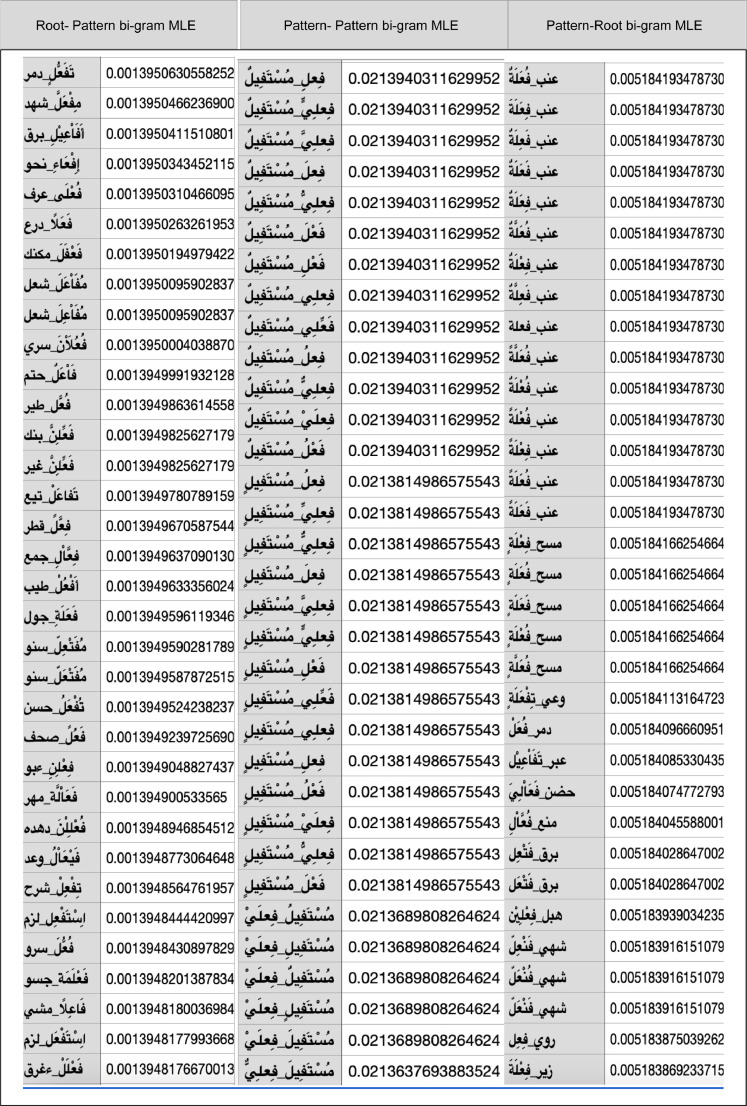
Sample of a fragemnt of P(root|pattern), P(pattern|pattern), and P(pattern|root) MLE. See page 13.

Fig. 6 shows a sample of a fragment of P(root|pattern), P(pattern|pattern), and P(pattern|root) MLE. Content: *The table contains multiple records descripting the result of the statistical analysis and their estimations. For example, the first record shows the MLE estimation of the probability that the root: DMR given the pattern “taC_1_aC_2_C_2_uC_3_ii” is 0.0014; i.e., P(DMR| taC_1_aC_2_C_2_uC_3_ii)=0.0014. Analog P(C_1_iC_2_C_3_i|mustaC_1_yC_3_uu) =0.0214 and so on*.

## 3 Experimental Design, Materials and Methods

**Fig. 7:**
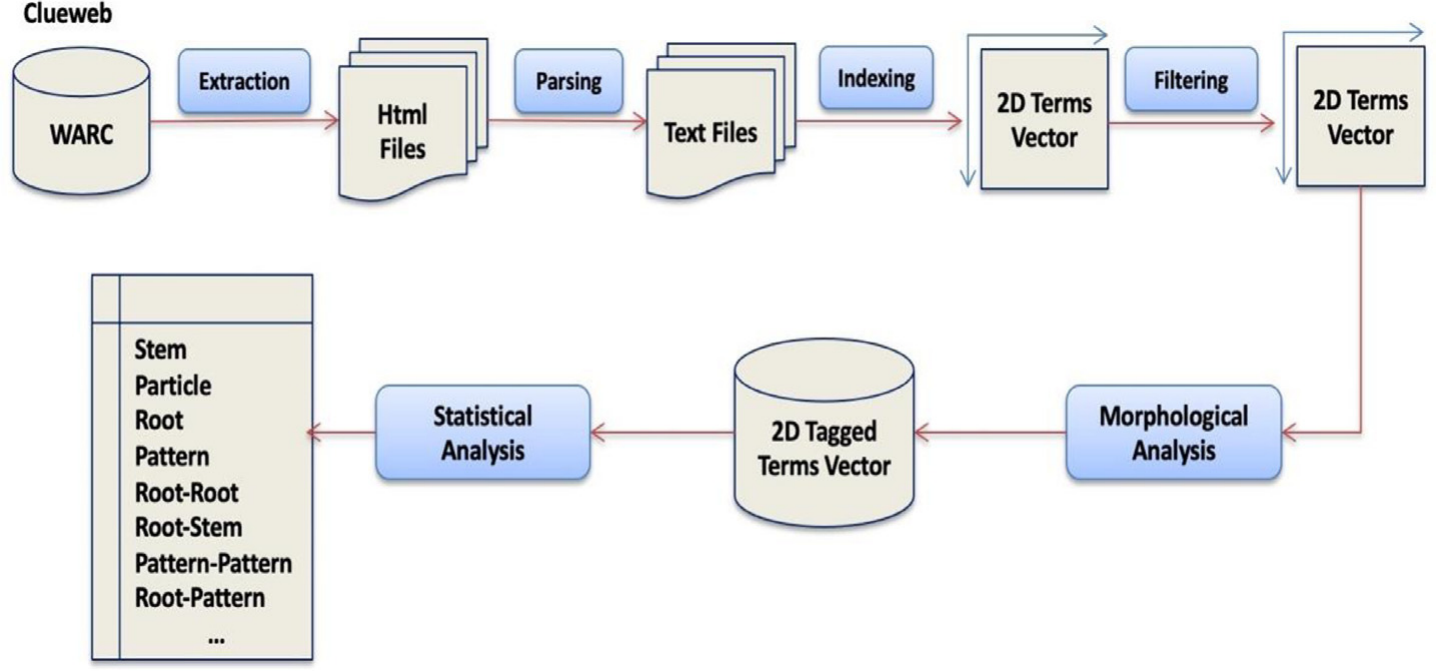
Data extraction and statistical analysis process.

The following processes were considered in the process of creating the Dataset see (Fig. 7).

• **Html files extraction**
  • As the Clueweb database is organized in a set of WARC archive files (Web ARChive), these files were extracted.
• **Html files parsing**
  • The next step is to extract the plain text from the html files, and this is done using an html parser.
  • The html parsing process reads and loads an html file and provides a way to traverse the html DOM for further processing like cleaning, formatting, or text extraction.
  • The result is 974 text files with the size of about 180 GB.
• **Indexing**
  • stablishing a vector space of all the distinct terms of the parsed text and count the frequencies of each two consecutive terms.
• **Filtering**
  • Filter out non-Arabic terms which might still be remaining (like Persian words).
• **Morphological Analysis**
  • Arabic Textware and Alkalil morphological analyzers, and Khoja stemmer were used to analyze corpus terms; this should give us for each word all possible roots, stems and patterns.

Sample from the Morph output (see figure (Fig. 7).

**Table:** 

Word	Prefix	Stem	Suffix	Root	Pattern
منتديات	-	منتدي	ات	ندي	مُفْتَعْل
منتديات	-	منتدي	ات	ندي	مُفْتَعِل
منتديات	-	منتدي	ات	ندي	مُفْتَعْل
الموضوع	ال	موضوع	-	وضع	مَفْعُوْل
الموضوع	ال	موضوع	-	وضع	مُفْعَوَل
الموضوع	ال	موضوع	-	وضع	مُفَعْوَل
الموضوع	ال	موضوع	-	وضع	مَفْعُوْل
الموضوع	ال	موضوع	-	وضع	مُفْعَوِل
الموضوع	ال	موضوع	-	وضع	مُفَعْوِل


 

• About 18.4 million words were extracted, with an indicator to the source of the analysis for each word (Arab Textware, , Al-Khalil, or Khoja), see (Fig. 3). Content: *the first record, the word form “muntadiate”(forums, plural from) parsed in Prefix: al article, Stem: mundata (forum), Suffix: at, Root: NDY, Pattern: muC_1_taC_2_C_3_*, and so on.
• **Basic Counting**
  • Counting the occurrence of frequency of roots, stems, and patterns appearing in the morph database.
• **Root-Pattern, Root-Root, Pattern-Pattern Analysis**
  • To extract the root-pattern probabilities we need to directly use the data output to count the number of times a specific root-pattern combination appears in a word, then to calculate the conditional probabilities as MLE estimation based on Laplace smoothing.

## Ethics Statement

The authors declare that this presentation meets all ethical requirements.

## CRediT authorship contribution statement

**Bassam Haddad:** Conceptualization, Methodology, Software, Writing – original draft, Writing – review & editing. **Ahmad Awwad:** Methodology, Investigation, Writing – review & editing. **Mamoun Hattab:** Supervision, Methodology, Software. **Ammar Hattab:** Software, Validation.

## Declaration of Competing Interest

The authors declare that there is no known competing financial interests or personal relationships which have, or could be perceived to have, influenced the work reported in this article.

## Data Availability

Language Data Model for Arabic based Root-Pattern MLE (Bi-gram) Analysis (Original data) (figshare). Morphological Dataset (Original data) (figshare). Raw Text Corpus (Unfiltered partially cleaned Html Raw Text files) (Original data) (zenodo).
